# Melamine disrupts spatial reversal learning and learning strategy via inhibiting hippocampal BDNF-mediated neural activity

**DOI:** 10.1371/journal.pone.0245326

**Published:** 2021-01-11

**Authors:** Wei Sun, Yuanhua Wu, Dongxin Tang, Xiaoliang Li, Lei An

**Affiliations:** 1 Behavioral Neuroscience Laboratory, The First Affiliated Hospital of Guizhou University of Traditional Chinese Medicine, Guiyang, China; 2 Department of Pediatric, The First Affiliated Hospital, Guizhou University of Traditional Chinese Medicine, Guiyang, Guizhou, China; 3 Department of Neurology, The First Affiliated Hospital of Guizhou University of Traditional Chinese Medicine, Guiyang, China; 4 Department of Neurology, Jinan Hospital, Jinan, China; Nathan S Kline Institute, UNITED STATES

## Abstract

Although several studies showed adverse neurotoxic effects of melamine on hippocampus (HPC)-dependent learning and reversal learning, the evidence for this mechanism is still unknown. We recently demonstrated that intra-hippocampal melamine injection affected the induction of long-term depression, which is associated with novelty acquisition and memory consolidation. Here, we infused melamine into the HPC of rats, and employed behavioral tests, immunoblotting, immunocytochemistry and electrophysiological methods to sought evidence for its effects on cognitive flexibility. Rats with intra-hippocampal infusion of melamine displayed dose-dependent increase in trials to the criterion in reversal learning, with no locomotion or motivation defect. Compared with controls, melamine-treated rats avoided HPC-dependent place strategy. Meanwhile, the learning-induced BDNF level in the HPC neurons was significantly reduced. Importantly, bilateral intra-hippocampal BDNF infusion could effectively mitigate the suppressive effects of melamine on neural correlate with reversal performance, and rescue the strategy bias and reversal learning deficits. Our findings provide first evidence for the effect of melamine on cognitive flexibility and suggest that the reversal learning deficit is due to the inability to use place strategy. Furthermore, the suppressive effects of melamine on BDNF-mediated neural activity could be the mechanism, thus advancing the understanding of compulsive behavior in melamine-induced and other neuropsychiatric disorders.

## 1. Introduction

Melamine, a triazine heterocyclic chemical, has been widely used in various industries, including plastics, dyes, fertilizers and fireproof materials. It is also approved as food-content substance, but it cannot be used as an additive [1]. In September 2008, melamine was illegally added to foods to increase their protein content, resulting in the melamine-contaminated milk powder scandal. Considering that young children are relatively vulnerable to food contaminants, melamine-contaminated milk incident has raised concerns about melamine toxicity in recent years. However, the effect of melamine and its mechanism are still unclear.

Although the nephrotoxicity of melamine has been widely reported [2–4], there is limited information concerning its neurotoxicity [5, 6]. Previously, the neurotoxicity of melamine had been linked to dysfunction and neuropathological alterations in the hippocampus (HPC), a region known to be critically involved in spatial learning and memory. For instance, melamine induced pathological changes in hippocampal structure, such as neuronal loss and necrosis [7, 8]. The damage induced by melamine to neurons mainly occurred due to the formation of insoluble metabolites in cells [9] and oxidative stress [8, 10]. Spatial learning and memory was impaired by chronic and prenatal exposure, with the mechanisms of neurotoxicity varying depending on the treatment route and the age of subjects [11–13]. Continuous melamine feeding to young rats inhibited both presynaptic and postsynaptic glutamate transmission, thereby affecting hippocampal synaptic function, leading to behavioral inflexibility [12, 14]. Furthermore, alleviation of oxidative damage caused by melamine in hippocampal CA1 area is known to reverse learning and re-acquisition deficits [15, 16]. Our recent findings showed that a single dose infusion of melamine into hippocampus hindered spatial memory consolidation via reduction in the levels of NR1 and NR2B subunits of N-methyl- D -aspartate (NMDA) receptors (NMDARs) [17]. Since long-term depression (LTD), which weakened synaptic transmission to enhance reversal performance [18, 19], was suppressed following intra-hippocampal infusion [17], it is unknown whether cognitive flexibility was affected.

Navigation towards a goal in certain types of learning tasks can be accomplished by executing distinct cognitive strategies associated with specific regions of the brain [20–22]. Place strategies rely on the HPC, an area important for flexible integration of novel information in an extra-maze environment [23, 24]. Alternatively, the striatum mediates a habitual form of learning in which stimulus-response habits accrue in an incremental or gradual fashion [25]. Notably, the two brain systems operate independently, in parallel, to control the type of information learned [26, 27]. In particular, deregulation of HPC function following lesions, pharmacological manipulations [28, 29], or temporary inactivation of neural activity [22] are associated with greater preference for a striatum-dependent learning strategy on tasks that also can be solved by HPC-dependent learning strategy. These results are consistent with the hypothesis that one neural system may process information that is not useful when solving a task that is predominantly dependent on another system, thereby interfering with cognitive behavior [26, 30, 31]. However, it remains unclear whether interfering with the functioning of HPC by acute melamine exposure disrupts spatial phenotype associated with facilitating response-based behavioral process.

In this study, we sought to determine whether the infusion of melamine into HPC affected reversal learning in Y-maze task. To better understand the action of melamine on reversal performance, the action of melamine on learning strategy was assessed in cross-maze task. Previously, the hippocampal BDNF level was increased by exposure to a novel environment [32]. Learning-induced expression of BDNF in the HPC was implicated for effective solving of maze task by place strategy [33–35]. Similarly, intrahippocampal infusion of BDNF facilitates strategy shifting by minimizing response perseveration to the previously acquired strategy [36]. Additional evidence has shown that the flexible memory system was associated with up-regulation of BDNF expression and transcriptionally permissive histone acetylation in the HPC [37]. Moreover, compelling evidence showed BDNF-mediated neural excitability verifying successful learning [38, 39]. Based on these findings, we further tested the effect of melamine on learning-induced BDNF expression and neural correlate of reversal performance. Our study helps to understand how melamine acts on reversal learning and place strategy, and the neural correlate of this cognitive process.

## 2. Materials and methods

### 2.1 Subjects

Male Sprague-Dawley rats (270–320 g, Beijing Research Center for Experimental Animals, Beijing, China) were individually housed in a temperature- and humidity-controlled room under a 12-h light-dark cycle (21±2°C; 45±5% humidity; lights on at 7:00 a.m.). Rats were restricted to maintain their weight at 85% to their as libitum weight with free access to water throughout the experiment in preparation for the Y-maze, T-maze and level press tests. Two weeks before the experimental day, rats were handled extensively (around 5 min per day). Experiments were conducted during the light period (between 2 p.m. and 5 p.m.) and experimenters were blind to the treatment of the animals. All animal experiments and procedures were reviewed and approved by the Experimental Animal Care Committee of Guizhou University of Traditional Chinese Medicine (SCXK-2013-0020).

Rats in the melamine groups were bilaterally infused with melamine (200 mM/μL or 400 mM/μL; Yingda Sparseness and Nobel Reagent Chemical Factory, China). Mel+BDNF group was injected recombinant human BDNF (1.5 μg/μL; St. Louis, MO, USA) into HPC 15 min following melamine infusions. Rats in the control and BDNF groups were received with artificial CSF (ACSF) and BDNF infusions into the HPC, respectively. The infusion was maintained at a rate of 0.5 μL/min for 2 min. According to previous studies [40–42], the dose was chosen as about 5 fold of the equivalent dose of human tolerable daily intake (TDI) of melamine, which was recommended by US Food and Drug Administration (FDA) in 2008 [43]. The dosage was converted from human dose to rat dose included height, weight and surface area by the online FDA Dose Calculator [44] and was corrected by conversion factor as previously described [45].

**Fig 1:**
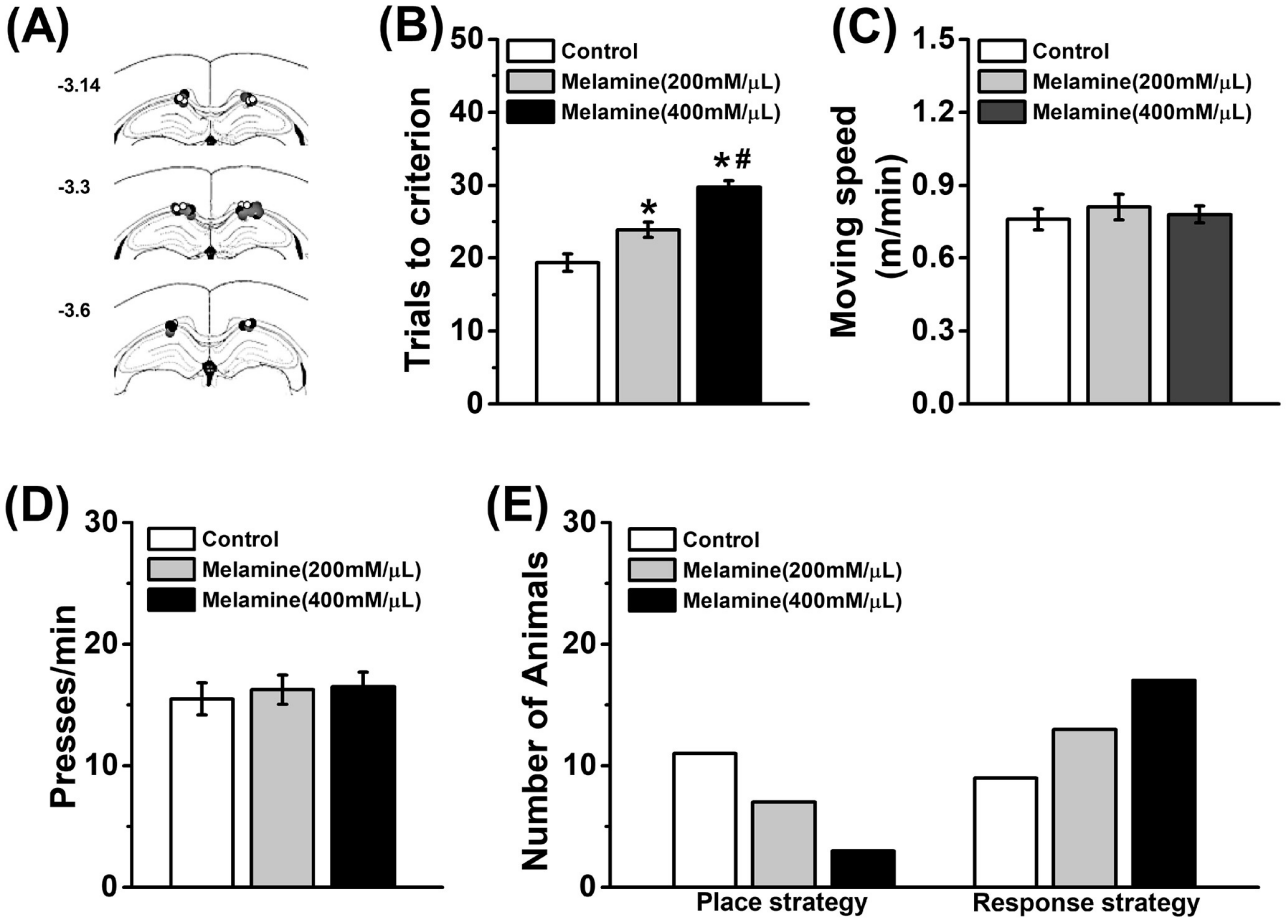
Intra-hippocampal melamine impairs reversal learning and cognitive strategy. **(A)** Schematic representation of the cannulae placements presented for the low dse (200 mM/μL) of melamine group (gray), for the high dose (400 mM/μL) of melamine group (black) and or the control group (hollow). **(B)** The total trails to the criterion in the reversal learning of Y-maze task. Both low and high dose of melamine induced reversal impairment, with a dose-dependent manner. *n* = 8 per group. Rats infused with melamine did not affect locomotion in open field task **(C)** or motivation behavior in level-press task **(D)**. *n* = 6 per group. **(E)** Learning strategy was tested in a probe trial of the cross-maze task and the number of rats that used each learning strategy was presented. Melamine-treated rats showed a learning strategy preference but avoid using place strategy. *n* = 20 per group. *P<0.05, vs. Control group; #*P*<0.05, vs. Melamine (200 mM/μL) group.

**Fig 2:**
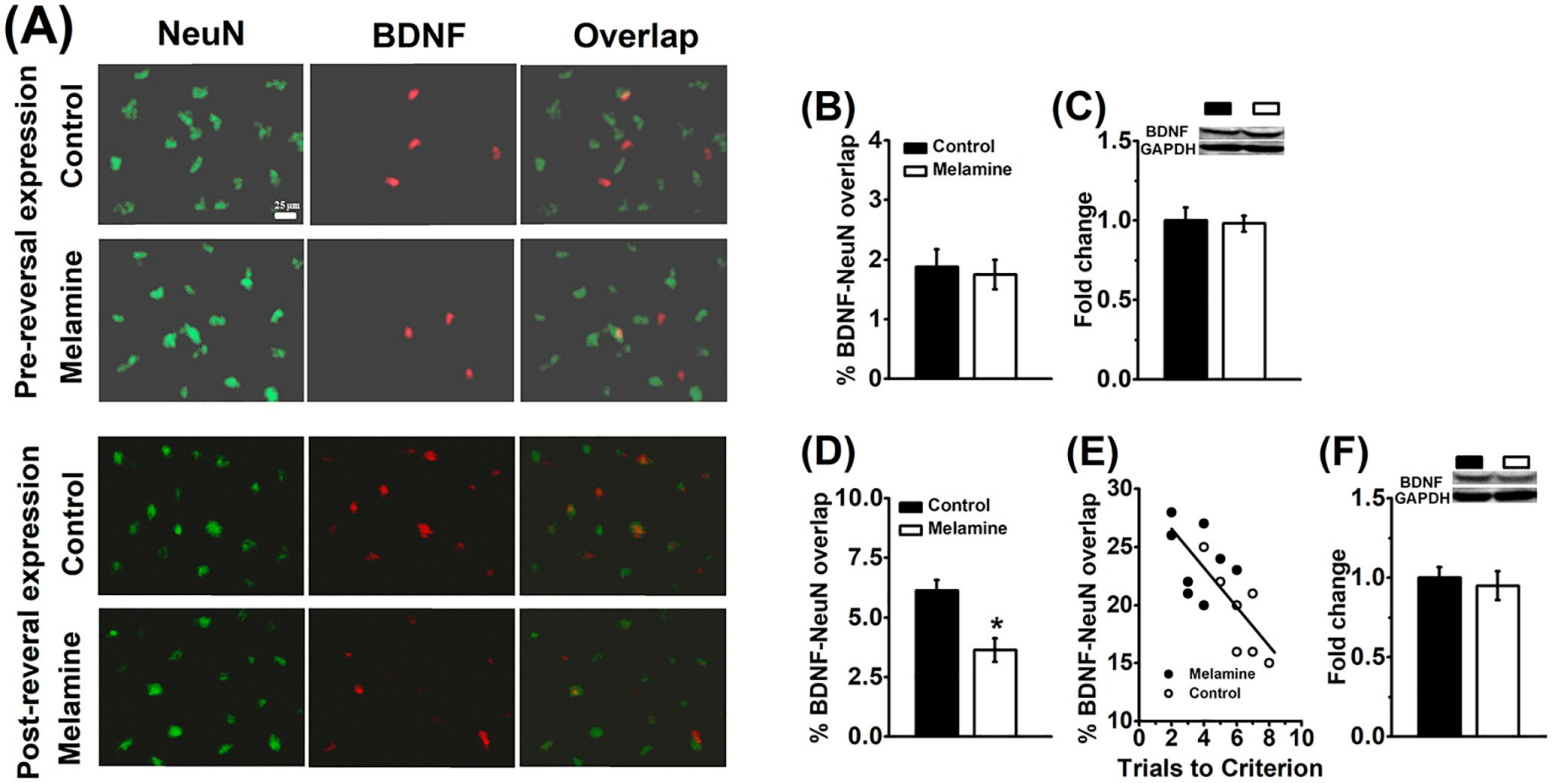
Melamine reduces learning-induced BDNF expression in the HPC neurons. **(A)** Representative micrographs showing labeling of NeuN (green), BDNF (red), and NeuN/BDNF overlap (yellow) in pre-reversal (Top) and post-reversal (Bottom) trained rats. After training in the learning stage of Y-maze task, the basal levels of BDNF in the neuron **(B)** and total BDNF **(C)** were tested 30 min following melamine treatment. No statistical difference was found before the reversal learning. *n* = 8 per group. **(D)** The BDNF level in the HPC neurons was significantly lower compared melamine to control groups. *n* = 8 per group. **(E)** A strong correlation between the trails to criterion during reversal learning and hippocampal BDNF expression. *P<0.05, vs. Good group. *n* = 8 per group. **(F)** After the reversal learning, there was no statistical difference in the total BDNF expression between melamine and control groups. *n* = 8 per group. *P<0.05, vs. Control group.

**Fig 3:**
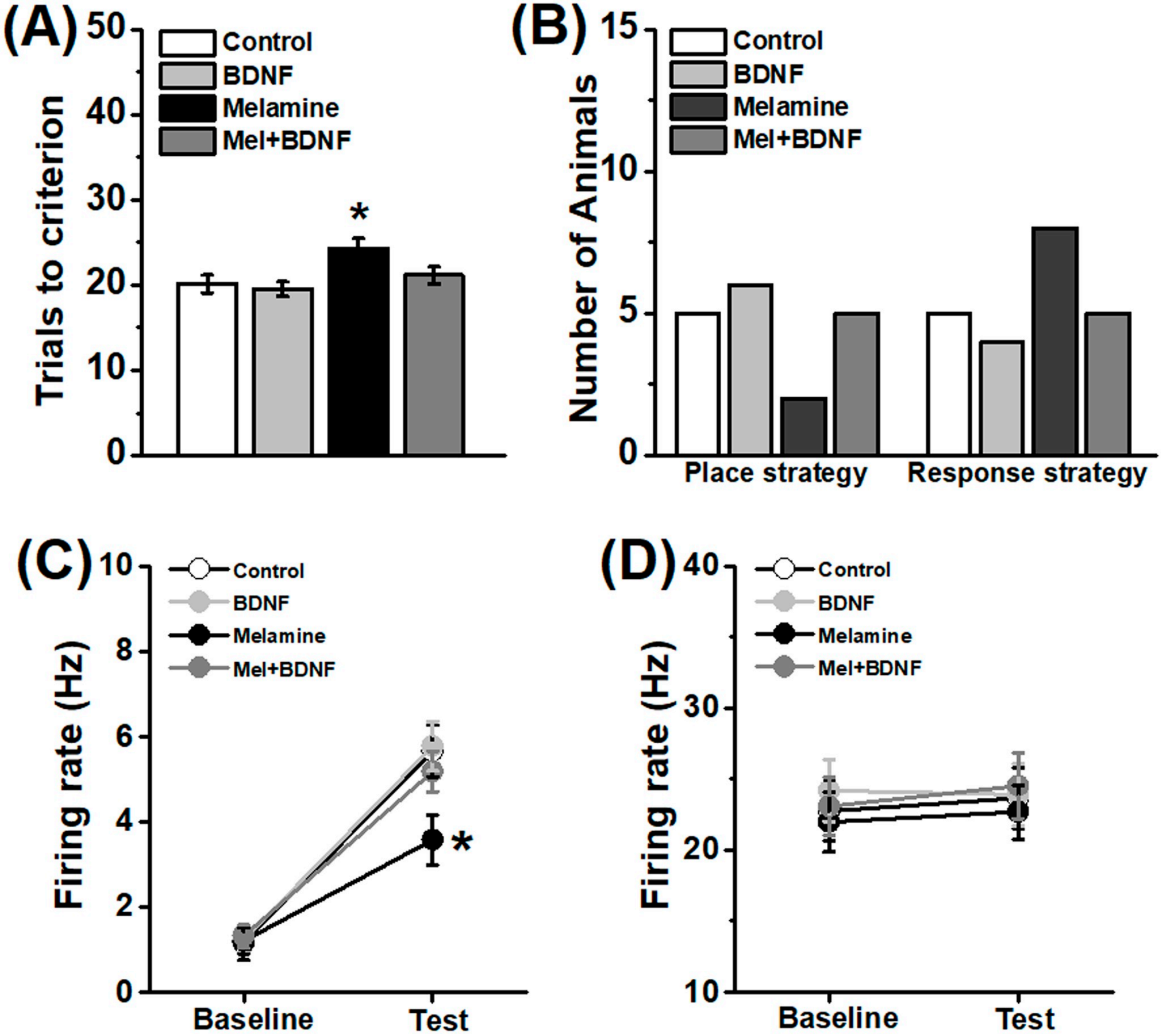
Intra-hippocampal BDNF infusion mitigates the disruptive effects of melamine on neural correlate of reversal performance and cognitive strategy. **(A)** Exogenous BDNF effectively reduced the total trials to the criterion in the reversal learning of Y-maze task. *n* = 8 per group. **(B)** Infusion BDNF into the HPC of melamine-treated rats could rescue the learning strategy bias. *n* = 10 per group. BDNF-mediated neural activity of pyramidal neurons **(C)**, but not FS interneurons **(D)**, was inhibited by melamine but reversed following BDNF treatment. *P<0.05, Melamine group vs. other groups.

Totally, two hundred and four rats were used in this study. Briefly, twenty-four, eighteen, eighteen and sixty rats were used in the Y-maze test, open field test, press tests, and corss-maze test, respectively (Fig 1). There were 8, 6, 6 and 20 rats in the Y-maze, open field, level press, and corss-maze tests of each group, respectively. The Hippocampi from the rats that tested in the Y-maze test were collected and detected the neuronal BDNF levels (Fig 2A). Additionally, twelve rats (6 in the control and 6 the melamine groups) were used to confirm the basal BDNF expression (Fig 2D). Thirty-two and forty rats were used to test the reversal effect of BDNF in the Y-maze (with recording) and cross-maze tests, respectively (Fig 3). There were 8, and 10 rats in the Y-maze and corss-maze tests of each group, respectively. The number of rats in each group was also indicated in the figure legend.

### 2.2 Learning and reversal learning in Y-maze test

The maze consisted of three arms (40cm×15cm×8cm) separated with 120° angles and built of black Plexiglas. The experiment room contained various distal spatial cues. An attached start box built of black Plexiglas (18cm×14cm×14cm) was separated from the entry arm of the Y-maze by a removable blocker. Small brown bowls (4.5 cm in height, 9 cm in diameter) were placed 1 cm before the end of both test arms, preventing visual inspection for food presence from a distance. Only one of the test arms was baited with 0.5 g of chocolate chips (Milka; Kraft Foods) as the reward. A blocker located halfway down each arm could be operated manually from the experimenter’s position and was used to allow animals only one choice in each training trial. The initiation of each trial was toned (1 kHz) by an auditory instruction cue presentation.

The procedure was conducted as our previous studies with modification [46, 47]. Briefly, the rat was placed in the start arm and allowed to visit the end of one of reward arms. After reaching the end of an arm, the rat was returned to its home cage that served as the inter-trial box. After each trial, arms were cleaned with 70% alcohol and allowed to dry completely. The inter-trial interval was about 20 s. The start arm was pseudo-randomly selected in each trial but counter-balanced across rats of groups. A visit was defined as the animal placing all 4 paws in one test arm. Success in response learning was defined as reaching a criterion of six correct trials in a row. On the next day, the animals were then subjected to reversal training, in which the food reward was relocated to the previously unbaited arm. Following the reversal, the session continued until criterion was reached on the newly baited side (six consecutive correct trials). Total trials to reach criterion during learning and reversal learning and velocity to reach the reward cup were quantified.

### 2.3 Cross-maze task

Rats were trained according to the versions of the cross-maze task [46, 47]. Training was conducted in a black Plexiglas cross-maze consisting of 2 start arms (e.g., north and south arm) and 2 test (reward) arms (e.g., east and west arm). All 4 arms were 45 cm long, 14-cm width, 8-cm height. Arms diverged at a 90° angle from each other. A start Plexiglas box (18cm×14cm×14cm) with a blocker was connected to the start arm. Only one start arm was used during the training days, the other start arm was blocked by removable black Plexiglas barrier. Several clearly visible, distinctive cues were fixed in the experiment room. Rat was allowed to visit all available maze arms until it obtained reward. If a rat did not reach the reward within 3 min, it was placed in the inter-trial Plexiglas box (45cm×45cm×52cm). After reward consumption, the experimenter placed the rat in the inter-trial box for 10–20 seconds. After cleaning all test arms and re-baiting the same arm. For each individual animal, the reward location was fixed, however reward location pseudorandomly between subjects and treatments during each training day. Rats were trained till reaching the criterion (six correct trials in a row). They were then subjected to a probe trial to assess the learning strategy used during training. During the probe trial, the animals started from the opposite start arm while the original start arm was blocked. Rats were rewarded whatever the choice they made in probe test. The same maze and environment were used, thereby insuring common sensory, behavioral, and motivational experiences. The probe trial had two possible outcomes: (1) rats using a place (spatial) strategy would visit the arm that was baited during training, i.e., the same spatial location or (2) rats using a response strategy would make the same turn as they had done during training and would visit the other arm.

### 2.4 Open field test

Locomotor activity was assessed in a 20-min open field, which consisted of a 91.5×91.5×61 cm^3^ Perspex box with dark walls, as described previously [48, 49]. The field was divided into a peripheral region (within 15.25 cm of the walls) and central region (61×61 cm^2^) of approximately equal area. Locomotion was recorded using VersaMax Activity Monitoring System (AccuScan Instruments, Columbus, OH).

### 2.5 Level press test

Rats were trained to lever press for food pellets in standard operant chambers located inside sound-attenuating boxes (Med Associates, St. Albans, VT). The chambers contained two retractable levers located on either side of a central food trough. As previous studies [17, 47, 48], rats were trained daily 30-min sessions with one of two levers extended randomly when the cue light above the level was illuminated. The schedule was progressively changed according to the sequence fixed ratio (FR)-1, FR-15, FR-30, and finally FR-60. Rats were tested in a 30-min session till they reached 10 presses per min on FR-60.

### 2.6 Surgery and microinjection

Rats were anesthetized with isoflurane and placed in a stereotaxic frame (SN-3, Narishige, Japan) for surgery [13, 50, 51]. Stainless steel guide cannulae (22-Ga; Plastics One, Inc.) were bilaterally implanted to the dorsal CA1 region of the HPC (AP: -3.3 mm, ML: ±2.2 mm, DV: 2.4–2.8 mm). Obdurators (30-gauge, Plastics One Inc.) were inserted into guide cannula to prevent obstruction. Rats were allowed to recover for seven to ten days.

Infusions were performed by inserting custom needles (30-Ga, Small Parts Inc.) connected through PE-50 tube into an infusion pump (Harvard Apparatus), extended 1.0 mm pass the end of the cannulae. After infusions, the needles were left for 3–5 min to allow the diffusion of the drug. Dose and route of administration were chosen based on our and other previous studies [17, 40, 48, 49, 52, 53]. The infusion needles were left in place for 3–5 min to allow the drug to diffuse. On each drug treatment day, the treatments were reversed or counterbalanced designs. One week before the treatment, infusion procedure was habituated on four separate days. The infusion sites were identified with the aid of The Rat Brain in Stereotaxic Coordinates (1997, third edition). Only data from animals with correct implants were analyzed (Fig 1A).

### 2.7 Immunocytochemistry

Rats were killed by overdose of urethane and the hemispheres were post-fixed in 4% paraformaldehyde for an additional 2 h, and placed in 30% sucrose cryoprotectant solution for 48 h. After freezing, coronal sections (20-μm thick) were processed on a cryostat (Leica Microsystems). Sections were incubated overnight with sheep anti-BDNF antibody (1:500, Millipore Bioscience Research Reagents). After rinsing in phosphate-buffered saline, sections were incubated with a fluorescent donkey anti-sheep IgG (1:1000; Millipore Bioscience Research Reagents) conjugated with Alexa Fluor 647 (1:500; Abcam). Sections were rinsed again in PBS, blocked with 10% normal donkey serum NGS, Vector Laboratories), and incubated for 3 hours with a polyclonal mouse anti-neuronal nuclei (NeuN) antibody (1:100; Millipore Cooperation). After rinsing in phosphate-buffered saline, sections were incubated with a fluorescent donkey anti-mouse IgG conjugated with Alexa Fluor 488 (1:500, Millipore). Finally, sections were rinsed again in PBS, mounted with Fluor-Gel (Electron Microscopy Sciences).

Four rostrocaudal sections of each animal were used for analyzing double labeling using a fluorescence microscope (Olympus, BX61) equipped with a digital camera (Color view, SIS). Image pairs were acquired using the appropriate filter sets for green Alexa Fluor 488 or red Alexa Fluor 647 fluorescence, respectively for NeuN or BDNF labeling. Images were processed using commercial software (Metamorph Software v7.7, Molecular Devices) by digitally removing background luminescence and automatically determining the threshold. The percentage of overlapping area between NeuN and BDNF images (co-labeling) was determined as previous studies [35, 53, 54].

### 2.8 Western blot analysis

Rats were killed by overdose of urethane and hippocampi were bilaterally dissected and homogenized in ice-cold lysis buffer (pH 7.4) containing a cocktail of protein phosphatase and proteinase inhibitors (Sigma, MA, USA). The samples were centrifuged at 12,000×g and 4°C for 10 min and the supernatant were collected. Protein concentrations were detected by bicinchoninic acid assay (Bio-Rad Lab). Equal amount of proteins were resolved by 10–15% SDS-PAGE and then transferred onto PVDF membranes (Pall, Florida, USA) for immunoblotting. The membranes were blocked with 5% non-fat skimmed milk for one hour and incubated with the primary rabbit anti-BDNF antibody (1:5,000; Chemicon, Temecula, USA). Mouse anti-GAPDH (1:5000, Chemicon, USA) was used as an internal control. After three washes with TBST buffer (10 min in each), the membranes were incubated with horseradish-peroxidase (HRP)-conjugated secondary goat anti-rabbit or anti-mouse IgG (1:1000; Southern Biotechnology Associates, AL) incubated for one hour. After three washes with TBST buffer, immunoreactivity was detected by ECL Detection Kit (CWBIO, China).

### 2.9 Electrophysiology

Microelectrode array was custom built in a 4 by 8 matrix of tungsten wires (25 μm, California Fine Wires) in a 35-Ga silica tubing (World Precision Instruments). They were then attached via gold pins to an EIB-36-PTB board (Neuralynx, Bozeman, MT). The electrode tips were gold-plated to 200–600 kΩ measured at 1 kHz (NanoZ, White Matter LLC, Seattle, WA). Rats were anesthetized with isoflurane and prepared for surgery using previously reported procedures [49, 55, 56]. Electrode arrays were slowly lowered into HPC and the hemisphere was implanted randomly but counterbalanced between rats. A stainless steel wire served as ground electrode and was soldered onto a jewelers’ screw, which was threaded into the skull.

Electrophysiological data were acquired on a Digitalynx system (Cheetah acquisition software, Neuralynx). Unit signals were recorded via a HS-36 unit gain headstage (Neuralynx) mounted on animal’s head by means of lightweight cabling that passed through a commutator (Neuralynx). Unit activity was amplified (1000–10000 times), sampled at 32 kHz and band-pass filters at 600–6,000 Hz. To verify the stability of recording, unit activities were recorded for about 30 min before baseline recording. The mean firing rates during baseline and the reversal training were recorded. Data from only the last 6 trials of reversal learning and the basal session was selected for further analysis.

After experiments, electrolytic lesions (10μA current for 10s) were applied to identify the recoding sites with reference to The Rat Brain in Stereotaxic Coordinates (1997, third edition). Only data from rats with probes located were used.

Spike sorting was performed offline with SpikeSort 3D, using a combination of KlustaKwik, followed by manual procedure (Klusters software package). Multiple parameters (spike height, trough, and energy) were used to visualize the clustered waveforms (Fig 3A). Each cluster was then checked manually to ensure that the cluster boundaries were well separated, and waveform shapes were consistent with action potentials [57, 58]. Units were then graded for quality and classified as pyramidal neurons and fast-spiking interneurons as previously described [46, 47].

### 2.10 Statistical analysis

Data are expressed as mean ± SEM. All analyses were performed with Neuroexplorer, Matlab (MathWorks), SPSS 17.0 software and Statistica software. Statistical analysis was conducted using one-way or repeated analysis of variance (ANOVA), or binomial tests followed by Tukey’s post hoc test. For the comparisons of strategy use between groups, Pearson χ^2^ analysis was performed. Correlations of the region-specific overlapping levels were made using the parametric Bravais-Pearson’s correlation test. Further details can also be found on the respective figures/results section. Differences were considered statistically significant when *P*<0.05.

## 3. Results

### 3.1 Intra-hippocampal melamine injection impairs reversal ability and reduces the use of place learning strategy

As shown in Fig 1A, the placements of the cannulae were placed just above dorsal CA1 region of the HPC. To detect if melamine affected behavioral performance in a dose-dependent manner, low (200 mM/μL) or high (400 mM/μL) dose was injected 30 min before tests. In the Y-maze task, rats in both low-dose and high-dose groups reached the criterion on the reversal learning slower than control group (Fig 1B, one-way ANOVA, effect of treatment: *F*_(2,21)_ = 29.60, *P*<0.001; post-hoc, both *P*<0.05). The low-dose group reached the criterion significantly fewer trials than high-dose one (*P*<0.05). Similar with previous studies [13, 41], no statistical differences in running speed were found (Fig 1C, one-way ANOVA, effect of treatment: *F*_(2,15)_ = 0.23, *P*>0.05). Furthermore, this impairment was not driven by a change in motivation as all rats had a similar motivation behavior in press lever test (Fig 1D, one-way ANOVA, effect of treatment: *F*_(2,15)_ = 0.11, *P*>0.05). This experiment indicates that melamine impairs the reversal performance in a dose-dependent manner. However, it was uncertain whether melamine affected the learning mechanism and strategy to search the baited location. For this reason, rats were subjected to training in the cross-maze task, and testing in the probe test 30 min following the melamine infusion. Attentively, control rats did not exhibit strategy preference while both melamine-treated rats displayed a strong preference for response strategy (Fig 1E, binomial test, both *P*<0.05). Compared with low-dose group, high-dose group significantly reduced the number of rats that used the spatial strategy (Fig 1E, Pearson χ2 test, *P*<0.05). Overall, our findings indicate that melamine suppresses the use of a spatial strategy resulting in the reversal impairment in the place-related task.

To detect if olfactory function involved in solving the task, two probe sessions were conducted immediately after rats reached the criterion of the reversal learning. The first probe session assessed if the scent guided choice by removing the reward from the bowl on a trial. If the rat made a correct choice, the experimenter placed a reward in the bowl. The second probe was implanted to assess if the rats marked the bowl when they examined previously. Therefore, the bowl was replaced by new one. The rats’ performance was 100% accurate during either of these probe sessions (Table 1).

**Table 1:** The performance in the two probe sessions following the reversal learning.

Group	The accurate	
	The first odor probe	The second bowl probe
Control	100%	100%
Melamine (200 mM/μL)	100%	100%
Melamine (400 mM/μL)	100%	100%

Additionally, we also attempted to confirm the effect of melamine on learning behavior. However, melamine-exposed rats did not exhibited learning deficits (total trials to criterion, control: 17.13±0.44, melamine: 17.63±0.38). Actually, either strategy could be used to correctly locate the food reward in two choices tasks. A shift from a HPC-dependent to striatum-dependent learning strategy in melamine-treated rats might account for the fact that we did find an attenuated performance during reversal training. Although either of the two available strategies was sufficient to locate the food reward during training, there is a particular difference between the systems underlying these two strategies in terms of flexibility.

### 3.2 Melamine reduces learning-induced neuronal BDNF but not basal expression

Previous studies reported that spatial-related training induced BDNF expression [53, 59] and activated BDNF signaling in the HPC [39, 46, 49]. To confirm if the learning strategy shift induced by melamine was accompanied by changes in the BDNF level, we detected BDNF expression immediately after reversal training. Given that BDNF is expressed in microglia and astrocytes cells [60, 61], we sought to detect the neuronal BDNF levels of pre-reversal (Fig 2A-top) and post-reversal learning (Fig 2A-bottom), were indicated by the area of overlap between BDNF and NeuN, a marker of neuronal nuclei. Melamine had no obvious effect on the neuronal (Fig 2B, T-test, *t*_14_ = 0.2, *P*>0.05) or total (Fig 2C, T-test, *t*_14_ = 0.1, *P*>0.05) expression of hippocampal BDNF in the pre-reversal learning condition. Melamine group showed significantly less neuronal BDNF than control group following reversal training (Fig 2D, T-test, *t*_14_ = 4.0, *P*<0.01). A strongly negative correlation between trials to criterion and BDNF level was observed (Fig 2E, Bravais-Pearson test, *r* = 0.56, *P*<0.001). In the post-reversal condition, the total BDNF expression of melamine group was comparable with that of control group (Fig 2F, T-test, *t*_14_ = 0.2, *P*>0.05). Overall, these results indicate that infusion of melamine into HPC declines training-induced BDNF expression, but not basal, expression.

### 3.3 Exogenous BDNF reverses melamine-induced behavioral deficits and neural activity

Given training-induced BDNF was inhibited by melamine, BDNF was infused into HPC 15 min before melamine treatment and then rescue the strategy bias. Training itself increased neural excitability, which was regulated by BDNF, its cognate receptor, and other related candidate effectors [23, 49, 53]. Similarly, the deteriorated effects of melamine on reversal behavior were observed as evidenced by the increase in trials to criterion (Fig 3A, one-way ANOVA, effect of treatment: *F*_(3,28)_ = 47.93, *P*<0.001; post-hoc, melamine vs. others, all *P*<0.05) and abstain from using place strategy (Fig 3B, binomial test, *P*<0.05). However, rats that infused with BDNF used fewer trials to the criterion than melamine-treated rats (Fig 3A, *P*<0.05). Furthermore, the infusion of BDNF could effectively rescue the strategy bias induced by melamine administration (Fig 3B, Pearson χ2 test, *P*<0.05). To better understand the effect of melamine on the neural correlates of behavioral strategy, single-unit activity was assessed in the HPC when rats performed the last 6 trials in the reversal learning task. One hundred and thirty-six units were sorted (pyramidal neurons: 28 from control group, 32 from BDNF group, 29 from melamine group, 30 from melamine+BDNF group; FS interneurons: 4 from control group, 5 from BDNF group, 4 from melamine group, 4 from melamine+BDNF group). The basal firing rate of pyramidal neurons was comparable among groups. However, melamine markedly diminished the firing frequency of pyramidal neurons during the reversal task (Fig 3C, one-way ANOVA, effect of treatment: *F*_(3,115)_ = 36.59, *P*<0.001; post-hoc, melamine vs. others, all *P*<0.05). The infusion of BDNF resulted in a significant improvement (Fig 3C, *P*<0.05), even turn back to the normal level. No significant difference was found in the firing rate of FS interneurons (Fig 3D, one-way ANOVA, effect of treatment: *F*_(3,13)_ = 0.47, *P*>0.05). Together, these findings indicate that the infusion of BDNF into the HPC could reverse the disruptive effects of melamine on neural correlate of place learning strategy.

## 4. Discussion

The purpose of the current investigation was to assess whether intra-hippocampal melamine injection induces impairment of reversal learning and explore the underlying mechanism. Our findings demonstrate that the infusion of melamine into HPC leads to prefer response rather than place learning strategy and results in reversal disability. The melamine-stimulated down-regulation of learning-induced neuronal BDNF is attributed to the strategy bias. BDNF infused into HPC could mitigate the response-strategy preference and reversal learning deficits induced by intra-hippocampal injection of melamine. Meanwhile, the enhanced BDNF expression in the HPC mitigates the suppressive effect of melamine on firing rate of pyramidal neurons, but not FS interneurons, during the reversal test. Our findings for the first time provide evidence that melamine inhibits BDNF-mediated neural activity, which is closely related to the disruption of behavioral strategy and flexibility.

Melamine (500 μg/mL) impaired action potential properties are related to the modulations of both potassium and sodium channels [6]. Our previous findings showed that the inhibition of long-term depression induced by 200 mM/μL melamine was not attributed to neurotransmission dysfunction [17]. Furthermore, a higher dose of 500 μg/mL, but not 50 μg/mL, increased the paired-pulse ratio [40], which is thought to reflect the Ca^2+^-dependent probability of spike-dependent transmitter release. Similarly, Wang et al. reported that melamine (312 mM/mL) disrupted the homeostasis of Ca^2+^ [7]. Consistent with these findings, our results indicate melamine doses of 200 mM/μL and 400 mM/μL disrupt hippocampus-dependent cognitive and neural function. Specifically, the selected doses were also in the range of the contents that detected in the contaminated dairy food products in the local area [62].

Reversal learning can be understood as a simple form of behavioral flexibility, which is the ability to inhibit previously acquired association and learn the new choice. Our initial results provide evidence that melamine-treated rats had more difficulty learning the new reward arm, suggesting impaired reversal learning and a slower erasure of the previous memory. Similar to other findings, rats displayed no deficits in locomotor and motivation behavior [17, 40], but their performance deteriorated as there was a change in HPC-dependent reversal learning in the water maze task [14]. Mental rigidity, perseveration, inability to shift, to adapt and to adjust behaviors to the context are common features of synaptic function disorders such as schizophrenia, depression, stress and aging [63–65]. The precise neurocellular mechanisms are still debated, but activation of hippocampal NMDA receptors and the induction of multiple forms of NMDA-dependent synaptic plasticity appear necessary. Our previous findings indicated that melamine reduced the expression of NMDA-NR2B subtype and inhibited a form of hippocampal synaptic depression [17], which may contribute to spatial reversal learning and oppose behavioral perseveration [18]. Indeed, the action of LTD at particular synapses is not simply a forgetting mechanism but is also required for cognitive flexibility to simultaneously inhibit previously learned information and facilitate acquisition of new memory [66, 67]. Additionally, disturbing the calcium homeostasis [7] and presynaptic Ca^2+^ release [9, 17], which are considered to be involved in the selective action of melamine in suppressing the excitability of pyramidal neurons, and thereby learning, memory and other physiological processes. Interestingly, rats with bilaterally intra-hippocampal melamine infusions preferred to use an egocentric response strategy but not an allocentric place strategy. Intact animals can use either place or response strategies, showing an individual preference for one or the other when both strategies are effective. Actually, the most effective strategy is dependent on task demands [28, 29]. When either the dorsal stratum or hippocampus was lesioned, the behavior was operated by the undamaged system [22, 27]. In comparison with the HPC, the dorsal striatum generates more stereotypical and less flexible responses that are more difficult to adapt to changing conditions [30, 68]. In agreement with this, rats using a response strategy in a T-maze learning paradigm have more difficulties learning the novel location of the food reward during reversal training [69]. Thus, our results implied that after a place strategy or rule was learned during the previous learning phase, melamine ruined the expression of the place strategy in the reversal task, leading to impair reversal phenotype.

There is increasing behavioral evidence indicating that BDNF expression is induced in the HPC following contextual and spatial learning and that this mechanism is essential for normal learning and memory [39, 70]. Similarly, BDNF has been shown to contribute to neuronal activity-dependent processes [53, 71], indicating its role in memory consolidation [72, 73]. Notably, our data suggests that the behavioral effects of melamine may result from reduced training-induced BDNF in the HPC. BDNF can activate BDNF-TrkB (tyrosine kinase B receptor) signaling that increases the expression of GluR1 and GluR2/3 AMPA receptor subunits [74] and the phosphorylation of the NMDA receptors [75, 76], leading to enhance neuronal excitability. Consistent with this, BDNF Val66Met polymorphism resulted in reduced NMDA receptor neurotransmission in the CA1 pyramidal neurons and impaired memory extinction [77, 78]. Studies in rats have shown that the formation of memory is related to prolonged phosphorylation and activation of hippocampal CREB, which by binding to a critical Ca^2+^ response element within the BDNF gene activates BDNF transcription to modulate synaptic transmission [79, 80]. In a recent study, we showed that activation of PKA/CREB/BDNF signaling involves functional couplings between HPC and striatum in spatial learning processing and strategy selection [46, 47]. Altogether, the impairment of cognitive flexibility induced by melamine is due to the reduction in learning-induced BDNF levels in HPC neurons leading to inhibition of neural activity.

Although spatial memory acquisition depends critically on the HPC, prefrontal cortex (PFC) function is also required for this process [81]. Spatial information generated in the HPC is relayed to the PFC via the CA1-PFC projection [82, 83]. Training-induced BDNF expression may influence behavioral flexibility by controlling the flow of information from the hippocampal CA1 to the PFC [84]. It cannot exclude that PFC region plays an additional role in the reversal learning of melamine-treated rats. Given that disruption of this circuit may lead to impairments in reversal learning [83, 85], future experiments should examine whether a disrupted HPC to PFC information flow is linked to the deficits observed in melamine-treated animals.

In conclusion, our findings extend the understanding of the ffeffects of intra-hippocampal melamine on cognitive flexibility, which are mostly attributed to its disruptive effect on place strategy. The learning-induced BDNF expression has correlated with the trials to criterion observed in reversal learning, with a lower BDNF level in melamine-treated rats. Furthermore, the deteriorated flexible behavior could be attributed to the interference by BDNF-mediated neuronal excitability during reversal performance. This provided an important insight into the neurotoxicity of melamine and a potential new avenue for treatment of spatial learning deficit and related disorders.

## Supporting information

S1 Raw images (PDF)
