# Supplementary material for: Melamine disrupts spatial reversal learning and learning strategy via inhibiting hippocampal BDNF-mediated neural activity
Source: PLoS One. 2021 Jan 11;16(1):e0245326. doi: 10.1371/journal.pone.0245326 (PMC7799824; doi:10.1371/journal.pone.0245326)
Supplement: S1 Raw images — (PDF) [file pone.0245326.s001.pdf]

Supplementary information

Blot/Gel image data

Figure 2C and Figure 2F in the main text

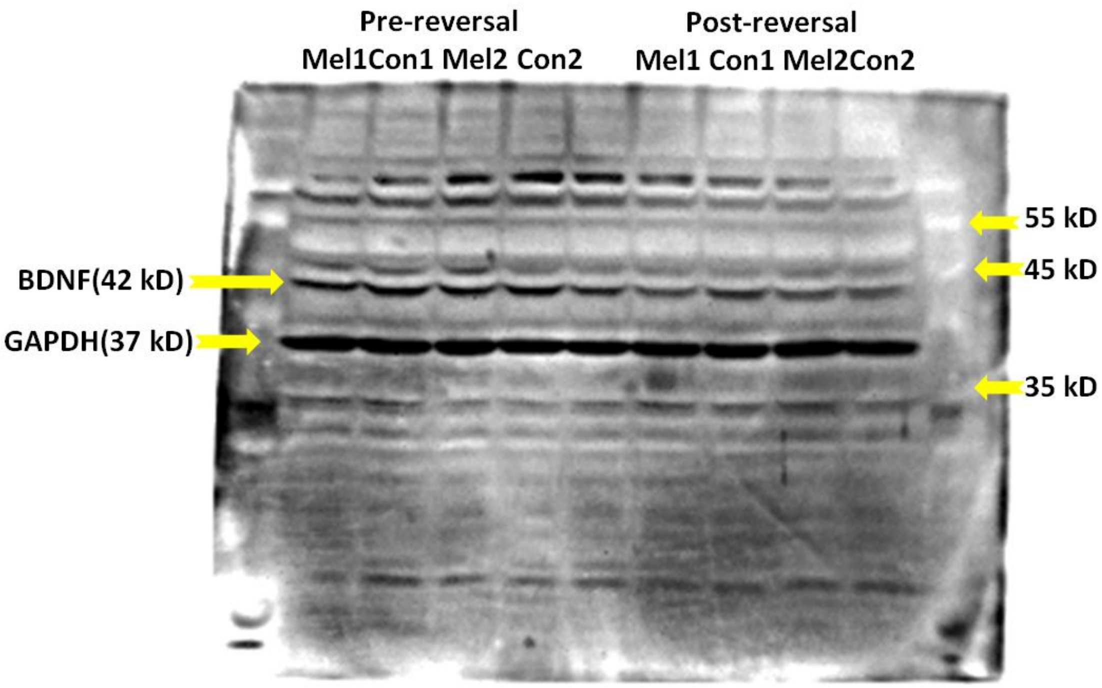

Note: Mel1 indicates a first sample of melamine group; Mel2 indicates a second sample of melamine group; Con1 indicates a first sample of control group; Con2 indicates a second sample of control group. Pre-reversal indicates the samples were collected before reversal training. Post-reversal indicates the samples were collected following the reversal training.
